# Spatial location and its relevance for terminological inferences in bio-ontologies

**DOI:** 10.1186/1471-2105-8-134

**Published:** 2007-04-20

**Authors:** Stefan Schulz, Kornél Markó, Udo Hahn

**Affiliations:** 1 Medical Informatics Department, Freiburg University Hospital, Freiburg, Germany; 2 Language and Information Engineering (JULIE) Lab, Jena University, Germany; 3 Master Program in Health Technology, Pontificial Catholic University of Paraná, Curtiba, Brazil

## Abstract

**Background:**

An adequate and expressive ontological representation of biological organisms and their parts requires formal reasoning mechanisms for their relations of physical aggregation and containment.

**Results:**

We demonstrate that the proposed formalism allows to deal consistently with "role propagation along non-taxonomic hierarchies", a problem which had repeatedly been identified as an intricate reasoning problem in biomedical ontologies.

**Conclusion:**

The proposed approach seems to be suitable for the redesign of compositional hierarchies in (bio)medical terminology systems which are embedded into the framework of the OBO (Open Biological Ontologies) Relation Ontology and are using knowledge representation languages developed by the Semantic Web community.

## 1 Background

Research activities in the "*omics*" sciences yield an ever-growing content of experimental data and publications, which are stored in a large variety of databases. For meta-data descriptions and mediation between these resources a large number of bio-ontologies have evolved. In a similar vein, medical terminology and classification systems have been developed in order to improve medical documentation and data analysis.

Whereas naive methods of ontology engineering (typically, the ad-hoc assembly of concept trees and graphs) were prevailing in the past, the claim is increasingly expressed for a more principled approach, based upon logics and formal ontology design methodologies. (We here understand by formal ontologies representational artifacts which use a formal language to describe entities and their relations in the domain of choice.) The need for taming the mass of newly generated domain knowledge requires sophisticated and rigid computational methods to support semantic interoperability on a large scale. This can only be fulfilled when discretionary modeling decisions and implicit semantic assumptions underlying the assembly of terms and relations are avoided, as much as possible.

This paper is structured in the following way. First we will address taxonomies and partonomies as the main hierarchical principles in the context of concrete instances of bio-ontologies. We will introduce relations considered canonical to relate entities in our domain. In this context we will focus on boundary problems related to parthood and location. The central part of the paper is dedicated to the so-called "role propagation" phenomenon, a reasoning pattern that is of high relevance for the domain. We will compare two approaches to represent this type of inferencing and provide recommendations for a parsimonious and ontologically adequate model.

## 2 Taxonomic Aspects in Bio-Ontologies

### 2.1 A Simple Upper Ontology

**Figure 1:**
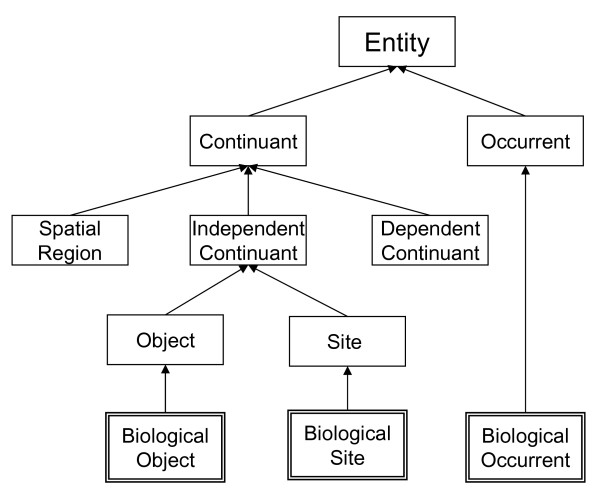
A simplified upper ontology for biology. Siblings are disjoint but not exhaustive.

In the following we focus on organisms and their physical components which are referred to as *Biological Objects*. According to a simplified upper ontology (see Fig. 1), which roughly follows the *Basic Formal Ontology *(BFO) [1,17], *Biological Objects *are classified as *Objects *that are *Independent Continuants*. According to BFO, *Continuants *are those entities in the world that persist through time by being present in their entirety at every point in time at which they exist. Organisms, populations, organs, cells, cell components, molecules, and atoms are examples for BFO *Objects*. BFO *Objects *as *Independent Continuants *are opposed to *Spatial regions *on the one hand, and *Dependent Continuants *on the other hand. (*Dependent Continuants *are further divided into *Qualities *and *Realizable Entities *such as functions, roles, and dispositions, which are not an issue in this paper.)

At the uppermost level, we distinguish *Continuants *from *Occurrents*. According to BFO, *Occurrents *are those entities in the world which change in time. This means that each portion of the time during which an occurrent occurs corresponds to a temporal portion of the occurrent, because occurrents exist only in their successive temporal parts, phases [16], or stages [9].

*Occurrents *are characterized by their ontological dependency on *Continuants*. For instance, every instance of the occurrent *Cell Differentiation Process *involves some instance of the continuant *Cell*.

As there is no uncontroversially accepted criterion for subdividing occurrents in terms of processes, events, changes, or actions, we do not make any further distinction.

Placing these categories into a context of biology, we end up with three disjoint (though not exhaustive) classes, *viz*. *Biological Site*, *Biological Object*, and *Biological Occurrent*, which are sufficient for our further considerations.

As familiar as the hierarchical arrangement of classes in a taxonomic framework may seem at the first sight, some clarification is necessary. First of all, the relationships between classes at the ontological layer and concrete individual entities in the world have to be characterized. We deliberately refrain from a principled discussion (which would have to cover nearly 2500 years of philosophy, beginning with Aristotle and addressing several controversial strands of philosophical thinking). We rather axiomatically introduce the relation **instance_of**, which relates an individual entity with the classes it belongs to. According to the OBO Relation Ontology [44], its domain and range do not overlap: No class can be **instance_of **anything, and individuals can never be instantiated. The division between classes and individuals is often treated in a fuzzy fashion, especially in naive approaches to ontology engineering. It may be tempting to describe, e.g. *Serotonin_Receptor *as **instance_of ***Protein *but then the representation of *Serotonin_Receptor_2A *would be impossible since an instance cannot be instantiated itself [33]. From a strict ontological point of view, even *Serotonin_Receptor_2A *would be a class (unless it is meant to refer to a single molecule or a defined amount of molecules, cf. [40]).

In line with the OBO Relation Ontology, we introduce the taxonomic subsumption relation *Is-A *by the following axiom:

∀*A*, *B *: (*Class*(*A*) ∧ *Class*(*B*) ∧ *Is*_*A*(*A*, *B*) =_*def *_∀*x *: (**instance_of**(*x*, *A*) ⇒ **instance_of**(*x*, *B*)))

This relation is equivalent to class subsumption in description logics [5] where the notation: A ⊑ B is used.

*Is-A *relates specific with more general classes and it constitutes the organizational principle of taxonomic hierarchies.

According to [44], classes and relationships between classes are represented by using *italic *font; we use **bold face **for all other relations.

### 2.2 Common Bio-Ontologies and their Architecture

The class *Biological Object *(as introduced above) occupies a prominent space in bio-ontologies, covering the whole range from biomolecules to organisms. The pivotal role of *Biological Object *is due to the fact that its descendants form the backbone of any principled bio-ontological approach. Apart from the fact that the study of their structural properties is of utmost importance in molecular biology and genomics, biological functions *inhere *in biological objects, biological objects *participate *in biological occurrents, e.g. in biochemical pathways, and they are the subject to a broad range of experimental or therapeutic manipulations in biology and medicine.

There are numerous examples for *Biological Object *ontologies such as

• The *Cellular Component *branch of the *Gene Ontology *(GO) [14], describing species-independent relations between cell components;

• The ontology of *Chemical Entities of Biological Interest *(ChEBI) [12];

• Many animal and plant anatomies hosted by the *Open Biological Ontologies *(OBO) platform [23];

• The *Foundational Model of Anatomy *(FMA) [31,32], which describes the canonical anatomy of the adult human, with a focus on macroscopic anatomy;

• The "anatomy" schema of the *GALEN CORE *model [2,29];

• The "anatomy" part of the *NCI Thesaurus *[18];

• The "anatomy" branch of *SNOMED CT *[3].

As a kind of unifying principle, the nodes in ontologies of biological objects are mostly arranged in a directed acyclic graph, using a bipartite hierarchical structure:

*Taxonomic links *associate specific with general classes using the relation *Is-A*, whereas *mereological links *[46] associate parts and wholes.

## 3 Mereological Aspects in Bio-Ontologies

### 3.1 The Meaning of "Part"

According to our stipulations, relations between parts and wholes require a principled account under the following aspects:

1. There must be a clear commitment to *formal properties*, i.e. in terms of transitivity, reflexivity and symmetry, as well as domain and range.

2. The wide-spread use of relations such as "part-of" and "has-part" between *classes *seems intuitive at a first sight, but is ambiguous and contrasts with the classical approach to mereology [43], which focuses on *individual entities*, and not classes of entities.

3. It has to be made explicit whether parts and wholes should be understood in a either functional or a purely locative sense and how temporal aspects are treated. The boundary to other relations should be made clear.

None of the ontologies of biological continuants mentioned above make sufficient claims regarding these three controversial issues.

FMA and GO have a clear commitment to item 1, at least regarding the transitivity property of the general "part-of" relation. The same applies to GALEN with regard to "part-of" subrelations [29]. This view is mainly consistent with classical (i.e., axiomatic) mereology [10,43] which treats *generic parthood *as reflexive, antisymmetric and transitive, with *proper parthood *as its irreflexive variant, excluding equality. It is the latter one which better fits the habitual understanding of "part-of" in biology. In contrast to this, the OBO Relation Ontology has recently introduced the "part-of" relation as a reflexive one [44].

Concerning other formal properties, such as extensionality, supplementation, or the treatment of time [7,9,43], none of the specialized biomedical terminology system takes up a defined position. With regard to the time aspect, the OBO Relation Ontology introduces the relation *derives-from *which includes the notion of what can be called *historic parthood*. Hence a DNA extracted from a cell is no longer "part-of" it. Implicitly, the mereological relations are restricted to continuants. Parts of occurrents are considered in *SNOMED CT *[3] but they are not identified as such [39].

Relating classes using mereological relations (item 2) is very common. However, semantic network style assertions such as *part-of*(*CellNucleus*, *Cell*), are so ambiguous that conflicting interpretations are likely to evolve: So did the Gene Ontology use to define this relation as follows: "*part-of *means *can be a part of*, not *is always a part of". *In the meanwhile this definition has changed to "The *part-of *relationship used in GO is usually (...) *necessarily is part*" [15]. In contrast, the Foundational Model of Anatomy (FMA) [32] uses class-level mereological relations in the following way: *part-of*(*A*, *B*) means that any instance of *B *has an instance of *A *as part and any instance of *A *is part of an instance of *B *[45] (This leads to problematic assertions such as *part-of*(*Rectum*, *Female Pelvis*)).

In this paper, we exclusively use mereological relations which hold between individual entities. Wherever they are used in class definitions quantifications will be used.

A rudimentary commitment to item 3 is reflected by the introduction of several mereological subrelations in FMA and GALEN [30]. For a more recent discussion of such subrelations, with a strong emphasis on biology, we refer to [8,21,26] who focus on the transitivity of mereological relations. According to [47], apparently plausible statements such as "*The nucleus of a cell is not a part of the organ which that cell is part of *" do not really challenge the assumption that the classical parthood relation is truly governed by formal ontological principles. It is rather the expression of an implicit narrowing of the sense of "part", which commonly happens in ordinary discourse. Taking the above statement, it holds true if we read "part" as "functional part", but not if we consider parthood in the broadest sense.

We here follow the OBO recommendations [44] and axiomatically introduce the relation **part_of **and its inverse **has_part **as transitive, antisymmetric, and reflexive relations which can be asserted either between pairs of continuants or between pairs of occurrents.

In addition we introduce the irreflexive parthood relations **proper_part_of **and **has_proper_part**. Parthood relations between classes are not considered because they can be defined in terms of **part_of **such as introduced in [44].

### 3.2 The Puzzle of the Parthood/Containment Distinction in Biology

**Figure 2:**
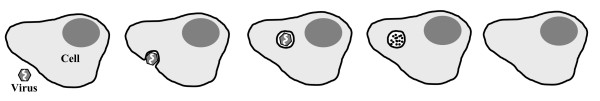
Phagocytosis of a virus by a cell: At which stage(s) one can state that components of the ingested virus are not only *contained *in but also **part_of **the cell?

**Figure 3:**
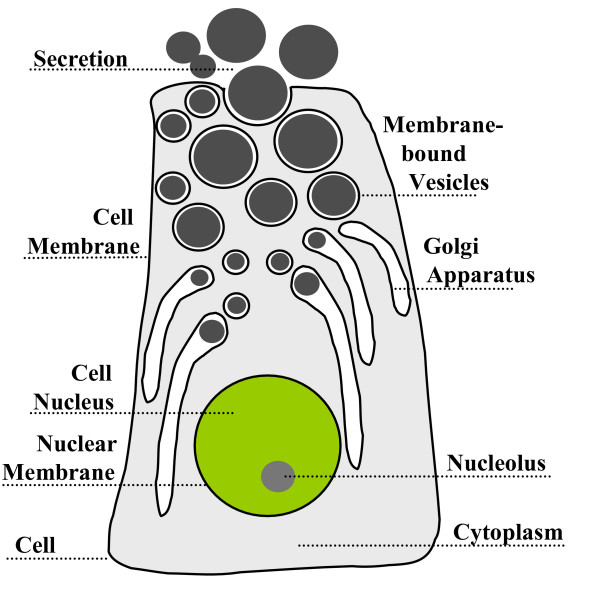
Prototypical Cell from a salivary gland: Are the secretions produced in the cell also **part_of **the cell or are they merely *contained *within them?

When we describe biological objects, it is often difficult to decide whether two objects are related in terms of parthood or in terms of a weaker relation of spatial inclusion. To a much higher degree than can be observed with artifacts, living biological entities continuously exchange matter with their environment. Typical examples are phagocytosis, pinocytosis, and exocytosis, such as depicted Fig. 2 and Fig. 3. In contrast to artifacts, there is no clear distinction between a system's constituents and its substrates. In contrast, as far as a mechanical engine is concerned, the material of which it consists is clearly distinct from the fuel it consumes and the exhaust-gas it produces.

Let us consider the following example: A carbon atom (e.g. as part of a carbohydrate) is ingested through the alimentary system where it can play quite different roles: It may merely participate in the organism's "power supply", after which it is eliminated by the lungs as part of a CO_2 _molecule or it may be integrated into the organism's constitutional structure, e.g. as part of a collagen fiber which then remains stable in the organism for years (cf. [35] for more examples of this kind). Other controversial examples on the cell level include small molecules and ions, as well as endosymbionts like mitochondria and chloroplasts. Another example is the reproduction process: A spermatozoon fuses with an oocyte then the male and female pronuclei form and merge. Afterwards the chromosomes are re-arranged, a zygote is formed and, finally, its cleavage is initiated. So it can be controversially discussed at which phase of this process which components of the original oocyte cease to be part of the female organism in which the oocyte has developed.

Organisms interact with other organisms as well. If a virus is in a cell, it is certainly not part of this cell. At which stage of the process of digestion (phagocytosis) do virus or bacteria components become parts of the ingesting cell (cf. Fig. 2) is difficult to delimit. Considering the cells which produce secretions (cf. Fig. 3), are these substances part of those cells or are they merely located within them? Many tissues such as the intestinal mucosa or the endometrium undergo permanent renovation, i.e. discharge of cells. Are these cells still part of the original tissue or not?

### 3.3 Spatial Location

In order to bring light into the parthood/containment dilemma, we introduce *location *as a new primitive for spatial aggregation of biological objects. Every instance of a continuant or an occurrent occupies a unique spatial region at every moment in time at which it exists. According to the Basic Inclusion Theory (BIT) [11] we introduce here the primitive **region_of**(*e*), a function of an individual entity *e*, the value of which is the spatial region *s *that *e *fully occupies. This corresponds to the region function *r *in BIT. In contrast to BIT, we here also allow occurrents to have a spatial location. We here define the region an occurrent fully occupies as the mereological fusion of all regions occupied by any stage (instantaneous part [9]) of this occurrent during its lifetime.

Using this notion of region, we define the relation **located_in **between two entities as follows: entity *x *is **located_in **entity *y *if and only if the region occupied by *x *is part of the region occupied by *y*.

∀*x*, *y *: **located_in**(*x*, *y*) =_*def *_**part_of**(**region_of**(*x*), **region_of**(*y*))

According to this, the **located_ in **relation holds between any two entities that occupy the same region. We do not discuss here whether it makes sense – according to our definitions – to talk about the location of an occurrent in another occurrent, or about an continuant in an occurrent. The only case we consider here concerns the location of a continuant in another one, or the location of an occurrent in a continuant. Location can also be asserted between regions since, according to BIT,

∀*x *: **region_of**(**region_of**(*x*)) = **region_of**(*x*)

We complete the picture by adding **location_of**, the inverse relation of **located_in**. Since the region occupied by a part of an object is always **part_of **the region occupied by the including whole, it follows that the relation **part_of **is a specialization of the relation **located_in**:

∀*x*, *y *: **part_of**(*x*, *y*) ⇒ **located_in**(*x*, *y*)

∀*x*, *y *: **has_part**(*x*, *y*) ⇒ **location_of**(*x*, *y*)

The relation **located_in **is further subdivided into **part_of **and **contained_in**. If an object is **contained_in **another one it is **located_in **it without being part of it. A distinction between **part_of **and **contained_in **is only possible for location between *Objects*, cf. [41].

∀*x*, *y *: **contained_in**(*x*, *y*) =_def _

**instance_of**(*x*, *Object*) ∧ **instance_of**(*y*, *Object*) ∧ **located_in**(*x*, *y*) ∧ ¬**part_of**(*x*, *y*)

It still remains difficult to distinguish parthood from location in an objective manner. In [41] we proposed a set of criteria which help decide whether a location relation between two objects *a *and *b *can be refined to a parthood relation. One criterion is the consideration of the objects' lifecycles. If *a *is always **located_in ***b*, then it can be regarded as **part_of ***b*. Another criterion we proposed is functionality. If *a *is **located_in ***b *and *a *functionally contributes to *b *then *a *is **part_of ***b*, at least if the function is affected when *a *is missing. Especially the latter criterion depends on an ontologically stable approach to biological function which is still due.

Finally, we introduce the primitive OBO relation pair **participant_of **and **has_participant**. This relation is asserted whenever biological objects are "participants" of an occurrent Unfortunately, OBO only vaguely characterizes this relation, namely "*a primitive instance-level relation between a process, a continuant, and a time at which the continuant participates in some way in the process. The relation obtains, for example, when this particular process of oxygen exchange across this particular alveolar membrane has_participant this particular sample of hemoglobin at this particular time*" [34]. We here understand the participation relation as a common subsumer of the 'agent' and 'patient' relations, as known from verb semantics [13].

Here, the agent is the instigator of an action, and the patient is the entity that undergoes any impact of an action or an event. We consider the relation **has_participant **in the context of biomedicine to relate a biological occurrent to those objects that are *directly involved *in it. For instance, a phosphorylation event has a kinase, a PO_4 _group, and an organic molecule as participants. Direct involvement has an important corrollary: The extension of participating objects is limited to the region where the occurrent is located. Therefore, if a single membrane protein is phosphorylated, neither the whole membrane nor the whole cell are considered participants of this phosphorylation event. In a similar vein, if a toe is amputated, the toe itself but not the whole foot acts as a participant.

**Figure 4:**
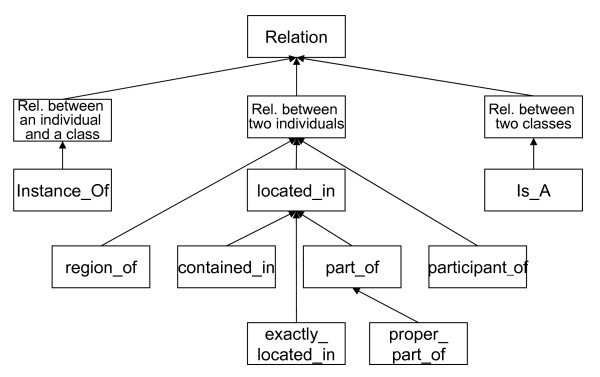
A simplified Relation Ontology for biology. Sibling relations are disjoint but not exhaustive.

Fig. 4 depicts the taxonomy of the relations which we use throughout this paper. Note that we have restricted ourselves to binary relations and that we ignore – for the sake of simplicity – the dimension of time. The relations follow the OBO suggestions, as introduced by [44], as well as the BIT, suggested by [11].

### 3.4 Patterns of Taxonomic and Partonomic Reasoning

We now address ourselves to the main topic of this paper, *viz*. the discussion how taxonomic and partonomic reasoning patterns interact and how plausible inferences can be drawn.

While reasoning in taxonomic hierarchies is relatively well understood – each instance of a specific class is also an instance of any more general class, and thus inherits all of its properties [24] – we lack an equal form of consensus for part/whole-related reasoning (cf. [4] for a survey).

The embedding of part/whole-related reasoning into more general mereological reasoning, i.e., the relation between parts and wholes and the space they occupy, are difficult issues in the construction of formal ontologies [10,11,38,41]. This is especially relevant when it comes to the representation of biological occurrents and their dependence on biological objects.

A frequently mentioned reasoning pattern is the so-called *role propagation *along mereological hierarchies. The importance of this problem is well accepted. It has led to the introduction of dedicated constructors in early knowledge representation languages, such as **transfersThru **in CycL [22] and **specializedBy **in GRAIL [28]. In a nutshell, role propagation is a reasoning phenomenon that is best explained by the following prototypical example:

"*Every instance of 'fracture of the neck of the femur' is also considered an instance of 'fracture of the femur' because every neck of a femur is a part of some femur*." [19]

Of course, this pattern is not restricted to reasoning about medical disorders and procedures but it extends to biology as well. So the biological occurrent of *Gastrin Secretion *is considered a process located at *Gastric G Cells *of the *Gastric Mucosa*, and therefore, we naturally classify it as a process located at some *Gastric Mucosa *since every instance of *Gastric G Cells *is part of some *Gastric Mucosa*.

In our previous research on biomedical knowledge representation [36-38,41] we have already addressed this issue extensively. We here provide a coherent account of modeling parthood in biological continuants in terms of spatial location. Based on the discussion of the nature of part-whole relations for biological continuants, we stipulate that for the purpose to support these inference patterns, the relations between wholes and their associated parts can be abstracted in terms of spatial relations, so that subsumption-based reasoning patterns can be reused to propagate roles across mereological hierarchies. The above reasoning pattern, which can be paraphrased as " *x, which is related to y, is related to z as well because y is a part of z*", is equivalent to the so-called right identity rule [6]:

∀*x*, *y*, *z *: **rel**(*x*, *y*) ∧ **part_of**(*y*, *z*) ⇒ **rel**(*x*, *z*)

The symbol **rel **stands for an arbitrary relation. This is instantiated by the following examples.

• "Glomerulonephritis is a kind of nephritis because glomerula are parts of kidneys and nephritis is an inflammatory disorder of the kidney."

• "Insulin production is usually considered a pancreas process, because pancreatic beta cells are located in the pancreas".

However, there are counterexamples:

• "An amputation of a toe is never a foot amputation, although every toe is part of a foot."

• "Mitosis is a cell process but it is generally not seen as a pancreas or liver process although these organs have cells as parts."

• "DNA replication cannot be not subsumed by cell replication although DNA is located in a cell."

This kind of anomaly make the usefulness of right-identity rules problematic [27], since there is ample evidence that such role inclusions do not always hold [38], as our examples suggest.

Let us analyze another example in a more principled way: Every instance of the class *Gastroenteritis *is an instance of *Inflammatory Disorder *related to some instance of *Digestive Tract *as a whole, and therefore, it is not identical with any instance of the class *Appendicitis*, which is defined as an *Inflammatory Disorder *of the *Appendix *– although every instance of *Appendix *is part of some instance of *Digestive Tract*. On the other hand, every instance of *Nephritis *is an instance of *Inflammatory Disorder *of some instance of either some *Kidney *or any of its parts. As a consequence, the class *Nephritis *subsumes *Glomerulonephritis *because every instance of *Glomerulonephritis *is an *Inflammatory Disorder *of some instance of *Glomerula*, each of which is part of some *Kidney*.

Obviously, some definitions relate to some entity type *as a whole*, whereas other ones relate to the *whole or any parts of it*.

In our previous work [37,38] we preferred to separately address the class of "a whole" (the so-called E-node), the class of "the proper parts of a whole" (the so-called P-node), and the class of "the proper or improper parts of a whole" (the so-called S-node).

**Figure 5:**
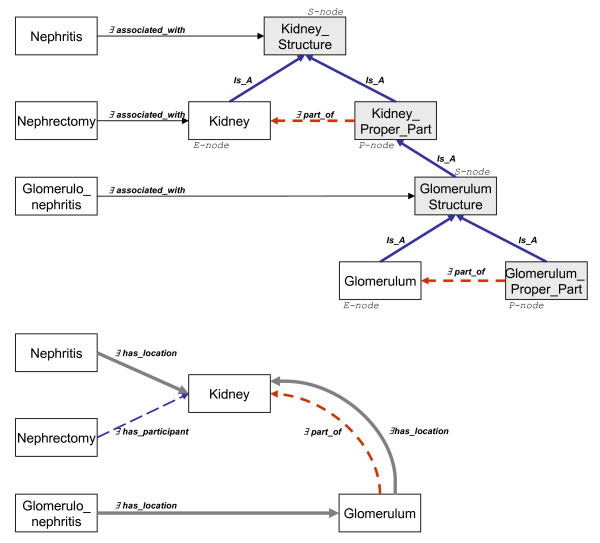
Top: SEP triplet approach for role propagation. Bottom: Alternative solution with semantically clear-cut relations. The relations labels use description logics [5] notation.

We argued that the introduction of artificial classes (which are defined as reifications of roles) is epistemologically valid, thus justifying the so-called SEP-triplets [42] approach. Fig. 5 (upper part) depicts a SEP-triplet model. Occurrents are linked to objects by a very general and not further defined relation *associated_with*. The burden of reasoning lies on the taxonomic structure. Since *Nephritis *is connected to the S-node class *Kidney Structure *and *Glomerulonephritis *is connected to the S-node class *Glomerulum Structure *which is subsumed by *Kidney Structure*, one can infer that *Glomerulonephritis *is a subclass of *Nephritis*. This reasoning pattern is not supported for *Nephrectomy *because here the target is *Kidney *as a whole and not a proper part of it. This approach was proposed in order to overcome the lack of former terminological languages which did not allow to directly specify algebraic properties of relations, such as transitivity or reflexivity. It has been taken up in the meantime by clinical terminologies, e.g. *SNOMED CT *[3].

However, the SEP approach is controversial mostly due to several facts. Firstly, it requires extensive modification of the class hierarchies. Secondly, the semantics of the extra nodes (S-Nodes and P-nodes) is frequently misunderstood. (In SNOMED CT, the same medical terms are assigned to E-nodes and S-nodes.) Finally, one can raise objections from a standpoint of formal ontology: The SEP model addresses (and solves) certain reasoning requirements in a superficial way, i.e. it does not require a formal analysis of the relations involved.

Furthermore, there are now terminological languages such as ℰ
 MathType@MTEF@5@5@+=feaafiart1ev1aaatCvAUfKttLearuWrP9MDH5MBPbIqV92AaeXatLxBI9gBamrtHrhAL1wy0L2yHvtyaeHbnfgDOvwBHrxAJfwnaebbnrfifHhDYfgasaacH8akY=wiFfYdH8Gipec8Eeeu0xXdbba9frFj0=OqFfea0dXdd9vqai=hGuQ8kuc9pgc9s8qqaq=dirpe0xb9q8qiLsFr0=vr0=vr0dc8meaabaqaciaacaGaaeqabaWaaeGaeaaakeaaimaacqWFWesraaa@3785@ℒ
 MathType@MTEF@5@5@+=feaafiart1ev1aaatCvAUfKttLearuWrP9MDH5MBPbIqV92AaeXatLxBI9gBaebbnrfifHhDYfgasaacH8akY=wiFfYdH8Gipec8Eeeu0xXdbba9frFj0=OqFfea0dXdd9vqai=hGuQ8kuc9pgc9s8qqaq=dirpe0xb9q8qiLsFr0=vr0=vr0dc8meaabaqaciaacaGaaeqabaqabeGadaaakeaat0uy0HwzTfgDPnwy1egaryqtHrhAL1wy0L2yHvdaiqaacqWFsectaaa@376D@, implemented in efficient reasoners, e.g. CEL [6] which support right-identity rules.

In the following, we propose an alternative solution to this problem where we completely refrain from complicated modeling artifacts. We show instead how the required inferences naturally derive from precise class definitions, using a parsimonious set of relations with well-defined algebraic properties. We further enhance our picture by extending the approach to disposition properties and their propagation along inclusion hierarchies.

## 4 A Formal Approach to Role Propagation across Partonomies

### 4.1 Integrating Taxonomic and Mereological Hierarchies

As we have shown in Section 3.1, the compositional structure of classes of biological objects cannot sufficiently be described by one single mereological hierarchy. As much as we can derive that an individual *b *has an individual *a *as part, knowing that *a *is part of *b*, we cannot transfer the same reasoning pattern to the level of classes.

Here, if we relate classes by quantified relations we can not infer that every instance of *B *has always some instance of *A *as part, knowing that every *A *is part of some *B*. (Using DL notation [5], *B *⊑ ∃**has_part**. *A *does not imply *A *⊑ ∃**part_of**.*B*.)

**Figure 6:**
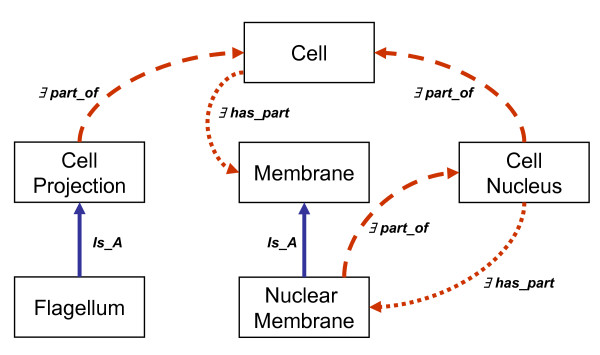
A complete mixed taxonomy/partonomy. In contrast to the OBO ontologies, *Part_Of *hierarchies here do not always coincide with *Has_Part *hierarchies. The relations labels use description logics [5] notation.

However, to the moment, the recommendation to treat links from the part to the whole differently from links that point from the whole to the part – although principally allowed at least according to the Gene Ontology usage guidelines [15]– has not been taken up by any of the OBO ontologies. Fig. 6 demonstrates how this might look like, using a couple of Gene Ontology classes. The partonomic relations between *Nuclear Membrane *and *Cell Nucleus *are bidirectional: every *Nuclear Membrane *is part of some *Cell Nucleus*, and every *Cell Nucleus *has some *Nuclear Membrane*. This is not the case with the classes *Cell*, *Membrane*, and *Cell Projection*. Not every *Cell *has a *Cell Nucleus *or a *Flagellum*, *Axon*, or other *Cell Projection*, but all of them are part of some *Cell*. Every *Cell *has some *Membrane*, but not every *Membrane *is part of some *Cell*.

These subtle though important partonomic distinctions have to be accounted for when we want to support plausible inferences involving biological objects and biological occurrents.

When we refer to the location of biological occurrents at a certain part of an organism or a cell we mean, more precisely, the location of this part. For this relation, we use the predicates **location_of **and **located_in**, in their broadest sense, including biological occurrents or dispositions: A biological object can therefore be the location of some biological occurrent, e.g. every instance of a biological occurrent *P *(e.g. *rRNA transcription process*) is located at some physical location *E *(e.g. *RNA Polymerase I*).

∀*x *: **instance_of**(*x*, *P*) ⇒ ∃*y *: **instance_of**(*y*, *E*) ∧ **located_in**(*x*, *y*)

### 4.2 Inferences involving Biological Occurrents

We now provide examples for plausible inferences, commonly referred to as propagation of properties.

#### Example 1

"A nephritis is an inflammatory disorder located at a kidney as a whole or at any of those objects which are necessarily part of a kidney."

∀*x *: **instance_of**(*x*, *Nephritis*) ⇔ **instance_of**(*x*, *InflammatoryDisorder*) ∧ ∃*y *: **instance_of**(*y*, *Kidney*) ∧ **located_in**(*x*, *y*)

The inference that every *Glomerulonephritis *is a *Nephritis *is then straightforward. The statement

∀*x *: **instance_of**(*x*, *Glomerulum*) ⇒ ∃*y *: **instance_of**(*y*, *Kidney*) ∧ **located_in**(*x*, *y*)

can be inferred from

∀*x *: **instance_of**(*x*, *Glomerulum*) ⇒ ∃*y *: **instance_of**(*y*, *Kidney*) ∧ **part_of**(*x*, *y*)

because **part_of**(*x*, *y*) ⇒ **located_in**(*x*, *y*). *Glomerulonephritis *is defined as:

∀*x *: **instance_of**(*x*, *Glomerulonephritis*) ⇔ **instance_of**(*x*, *InflammatoryDisorder*) ∧ ∃*y *: **instance_of**(*y*, *Glomerulum*) ∧ **located_in**(*x*, *y*)

Due to the transitivity of the relation **located_in **and the fact that every instance of *Glomerulum *is located in some *Kidney *(Formula 10), we get:

∀*x *: **instance_of**(*x*, *Glomerulonephritis*) ⇒ **instance_of**(*x*, *Nephritis*)

The relation **located_in **is used both for the spatial location of *Glomerulum *in *Kidney *and for the relation between the occurrent *Glomerulonephritis *and *Glomerulum*. So it is uniquely the transitivity property of the relation **located_in **which provides the required inference.

#### Example 2

*"Apoptosis is a kind of cell death. Tissue has cells as parts. Even though, apoptosis is not a necrosis (tissue death)"*.

∀*v *: **instance_of**(*v*, *Necrosis*) ⇔ **instance_of**(*v*, *Death*) ∧ ∃*w *: **instance of**(*w*, *Tissue*) ∧ **located_in**(*v*, *w*)

∀*x *: **instance_of**(*x*, *Apoptosis*) ⇒ **instance_of**(*x*, *Death*) ∧ ∃*y *: **instance of**(*y*, *Cell*) ∧ **located_in**(*x*, *y*)

The above statement would not necessarily hold if every instance of *Cell *were **part_of**, and consequently **located_in**, some instance of *Tissue*. This is not the case because there are cells which are not part of a tissue. However we could modify the example as follows:

#### Example 3

*"Neuron Apoptosis is a kind of cell death. Neural tissue has cells as parts. Even though, neuron apoptosis is not a necrosis of neural tissue"*.

Using the same inference as above we would, indeed, not be able to explain why apoptosis is not to be subsumed by necrosis. A closer look at Formula 14 reveals that "any death process located in some tissue" is a necrosis. Following this definition, of course, any isolated apoptosis event in a tissue would be equivalent to necrosis. This is certainly not what the term "necrosis" means. We will, therefore, have to correct the above definition, using the relation **participant_of **as introduced in Section 3.3:

∀*v *: **instance_of**(*v*, *Necrosis*) ⇔ **instance_of**(*v*, *Death*) ∧ ∃*w *: **instance of**(*w*, *Tissue*) ∧ **participant_of**(*w*, *v*)

For the proper definition of biological occurrents, as a general rule, we have to carefully distinguish between a biological object which is the *location *of an occurrent (i.e. the occurrent may occur even in a small part of this object), or biological objects which are *participants *of this occurrent, e.g. they are involved in the occurrent as wholes.

We underline this distinction by the following quite trivial medical example:

#### Example 4

*"Amputation ***of ***a foot is an amputation which targets a foot."*

∀*x *: **instance_of**(*x*, *Amputation Of Foot*) ⇔ **instance_of**(*x*, *Amputation*) ∧ ∃*y *: **instance of**(*y*, *Foot*) ∧ **participant_of**(*y*, *x*)

*"Amputation ***at ***a foot is an amputation which is located at a foot."*

∀*x *: **instance_of**(*x*, *AmputationAtFoot*) ⇔ **instance_of**(*x*, *Amputation*) ∧ ∃*y *: **instance of**(*y*, *Foot*) ∧ **located_in**(*x*, *y*)

As a consequence, any instance *AmputationOfToe *would be an *AmputationAtFoot *because every *AmputationOfToe *has some *Toe *as participant and every *Toe *is **located_in **some *Foot*, given the transitivity of the relation **located_in**. Note the subtle but important semantic distinction between the prepositions "at" or "in" (location) and "of" (participant).

The already discussed distinction in propagation patterns between *Nephritis *and *Gastroenteritis *can be explained by the same terms. Whilst *Nephritis *is an inflammatory disorder "at", *Gastroenteritis *is not only an inflammatory disorder "at" but also an inflammatory disorder "of". Therefore, *Glomerulonephritis Is-A Nephritis*, whereas *Gastroenteritis *does not subsume *Appendicitis*. This way, domain-specific specialized relations such as **inflammation-of**, **fracture-of**, **necrosis-of**, **amputation-of **(such as proposed in [42]) can easily be reduced to a small set of foundational relations, such as **participant_of **and **located_in**, an argument perfectly in line with the requirement of parsimony formulated in [44].

Finally, the propagation patterns fundamentally depends on the distinction between the direction of the class-level partonomic relation, such as illustrated in Fig. 6 and discussed in Section 4.1. This explains the reason why *Mitosis *is not a *Liver *process although every *Liver *has instances of *Cell *as parts. The explanation is that whereas for each instance of *Liver *there are instances of *Cell *and *Mitosis*, not every *Cell *is part of a *Liver*, and therefore not every instance of *Mitosis *is located at some instance of *Liver*.

### 4.3 An Attempt to Generalize

The two reasoning patterns, which had been accounted for in the SEP approach (cf. Fig. 5) by relating to the S-nodes (for enabling propagation) or to the E-nodes (for obviating propagation) can be reconstructed as follows.

First of all, we reinterpret the additional nodes in a straightforward way. The P-nodes for parts are axiomatized as reificators of the relation **proper_part_of**:

∀*p *: **instance_of**(*p*, *X*_*P*_) ⇔ (∃*x *: **instance_of**(*x*, *X*) ∧ **proper_part_of**(*p*, *x*))

while the S nodes as reificators of the relation **part_of**, formalized as follows:

∀*s *: **instance_of**(*s*, *X*_*S*_) ⇔ (∃*x *: **instance_of**(*x*, *X*) ∧ **part_of**(*s*, *x*))

The subsumption of P-nodes by S-nodes can be deduced from the fact that **proper_part_of **implies **part_of**.

As the relation **part_of **implies **located_in**, we obtain the following implication for P-nodes:

∀*p *: **instance_of**(*p*, *X*_*P*_) ⇒ (∃*x *: **instance_of**(*x*, *X*) ∧ **located_in**(*p*, *x*))

and for the S nodes accordingly:

∀*x *: **instance_of**(*s*, *X*_*S*_) ⇒ (∃*x *: **instance_of**(*x*, *X*) ∧ **located_in**(*s*, *x*))

Consequently, by using the same relation **located_in **to relate an occurrent with a continuant (instead of using the ill-defined relation **associated_with**), we obtain the conclusion that an occurrent located at anything subsumed by *X*_*S*_, *viz*. an occurrent located at any part of some *X *is located at some *X *due to the transitivity property of **located_in**.

Thus, the propagation of properties is a natural consequence of generalizing the notion of "location". If a process *p *is **located_in ***x *and *x *is **located_in ***y *(which is the consequence of *x *being **part_of ***y*), then the process *p *is **located_in ***y*, as well. Coming back to the example in Fig. 5, every instance of *Glomerulonephritis *is **located_in **some *Glomerula*, all *Glomerula *are part of some *Kidney *and therefore they are **located_in **some *Kidney*. Due to the definition of *Nephritis *as a disorder **located_in **some *Kidney*, we get the terminological inference that every *Glomerulonephritis *is a *Nephritis*.

How can we deal now with the "anomalous" cases? A closer look at these cases reveals that they always target some object as a whole. For instance, *Nephrectomy *is the removal of the whole kidney, while any partial removal of kidney tissue, e.g. in a biopsy, must not be classified as *Nephrectomy*.

Now the OBO relation **participant_of **comes into the play. As shown in Fig. 5, the node denoting a disease class is linked by this relation to the node denoting the class of biological objects. Again, we have to substitute the fuzzy relation **associated_with **relation by a canonical one. Since **participant_of **is not a subrelation of **located_in**, there is no inference pattern as with the *Glomerulonephritis *case.

One might still have reservations against the use of the relation **participant_of**, basically due to the lack of a clear definition. In this case, a solution would be to introduce the relation **exactly_located_in**, according to [10], which is defined as the exact coincidence of two regions.

∀*x*, *y *: **exactly_located_in**(*x*, *y*^) ^=_*def *_(**region_of**(*x*) = **region_of**(*y*))

Thus, we continue arguing on the level of spatial relations, without any hidden assumptions which may occur when using an ill-defined relation such as **participant_of**. Since **exactly_located_in **is a stricter relation than **located_in**, the role propagation is obviated. The effect is the same, and the use of **exactly_located_in **might indeed be preferred due to its semantic clarity. On the other hand, **participant_of **has been proposed as a foundational relation for bio-ontologies, so that there are good reasons to choose this one. However, this relation is still fuzzy and a substantial clarification of its semantics is due.

## 5 Conclusion

This paper deals with two major issues. The first one is concerned with ontological considerations underlying the substitutability of part-whole by locational relations, in accordance with the OBO (Open Biological Ontologies) Relation Ontology. For physical domains at least, it seems evident to get rid of the ongoing debate about different forms of part-whole reasoning and its underpinnings by abstracting away from **part_of/has_part **and describing the composition of physical objects in terms of locational relations, *viz*. **located_in **and its inverse **location_of**.

The second issue has to do with typical inferences drawn in the biomedical domain, *viz*. the propagation of attributes across partonomies. We here propose a straightforward solution where the relation pair **located_in/location_of **is used not only to express the spatial relationships between biological objects and spaces but also between occurrents and biological objects. Further work should extend this framework to include biological functions, qualities and dispositions [20,40], as well.

Thus we do refrain from additional formal language constructs in order to obtain the desired inferences (propagation of properties across compositional hierarchies) which are needed in such a reasoning framework. This way, we combine ontological clarity with formal simplicity and descriptional parsimony. We have used first order logics for our formalisms but it can easily be demonstrated that our use of FOL expressiveness translates into OWL-DL [25], the description logics [5] standard of the Semantic Web. The chosen approach is currently used for a principled redesign of the anatomy part of *SNOMED CT *[3]. There is preliminary evidence that newly designed terminological reasoners [6] are able to efficiently deal with such structures, so that the previous SEP triplet approach – introduced mainly for the purpose to enable efficient computation – can be abandoned.

## Authors' contributions

The method described was principally developed by SS. KM and UH participated in the formalization. All authors read and approved the final manuscript.
